# Transformation of Seed Non-Transmissible Hop Viroids in *Nicotiana* *benthamiana* Causes Distortions in Male Gametophyte Development

**DOI:** 10.3390/plants10112398

**Published:** 2021-11-06

**Authors:** Lenka Steinbachová, Jaroslav Matoušek, Gerhard Steger, Helena Matoušková, Sebastjan Radišek, David Honys

**Affiliations:** 1 Institute of Experimental Botany of the Czech Academy of Sciences, Rozvojová 263, 165 02 Prague 6, Czech Republic; steinbachova@ueb.cas.cz; 2 Biology Centre of the Czech Academy of Sciences, Department of Molecular Genetics, Institute of Plant Molecular Biology, Branišovská 31, 37005 České Budějovice, Czech Republic; jmat@umbr.cas.cz (J.M.); ilona@umbr.cas.cz (H.M.); 3 Institut für Physikalische Biologie, Heinrich Heine University Düsseldorf, D-40204 Düsseldorf, Germany; steger@biophys.uni-duesseldorf.de; 4 Slovenian Institute of Hop Research and Brewing, Cesta Žalskega tabora 2, SI-3310 Žalec, Slovenia; sebastjan.radisek@ihps.si

**Keywords:** male gametophyte, CBCVd, AFCVd and PSTVd parasitic RNAs, *Nicotiana benthamiana*, *Humulus lupulus*, plant transformation, proteomic of viroid infected pollen, viroid elimination

## Abstract

Viroids are small, non-coding, parasitic RNAs that promote developmental distortions in sensitive plants. We analyzed pollen of *Nicotiana benthamiana* after infection and/or ectopic transformation with cDNAs of citrus bark cracking viroid (CBCVd), apple fruit crinkle viroid (AFCVd) and potato spindle tuber viroid (PSTVd) variant AS1. These viroids were seed non-transmissible in *N. benthamiana*. All viroids propagated to high levels in immature anthers similar to leaves, while their levels were drastically reduced by approximately 3.6 × 10^3^, 800 and 59 times in mature pollen of CBCVd, AFCVd and PSTVd infected *N. benthamiana,* respectively, in comparison to leaves. These results suggest similar elimination processes during male gametophyte development as in the *Nicotiana tabacum* we presented in our previous study. Mature pollen of *N. benthamiana* showed no apparent defects in infected plants although all three viroids induced strong pathological symptoms on leaves. While *Nicotiana* species have naturally bicellular mature pollen, we noted a rare occurrence of mature pollen with three nuclei in CBCVd-infected *N. benthamiana*. Changes in the expression of ribosomal marker proteins in AFCVd-infected pollen were detected, suggesting some changes in pollen metabolism. *N. benthamiana* transformed with 35S-driven viroid cDNAs showed strong symptoms including defects in pollen development. A large number of aborted pollen (34% and 62%) and a slight increase of young pollen grains (8% and 15%) were found in mature pollen of AFCVd and CBCVd transformants, respectively, in comparison to control plants (3.9% aborted pollen and 0.3% young pollen). Moreover, pollen grains with malformed nuclei or trinuclear pollen were found in CBCVd-transformed plants. Our results suggest that “forcing” overexpression of seed non-transmissible viroid led to strong pollen pathogenesis. Viroid adaptation to pollen metabolism can be assumed as an important factor for viroid transmissibility through pollen and seeds.

## 1. Introduction

Pollen developed as highly synchronous generative tissue producing sperm cells and plays a specialized role in double fertilization in angiosperms [1]. Bicellular pollen, for example in *Nicotiana* sp. and *Humulus lupulus* [2], produces two sperm cells by division of generative cell later in the growing pollen tube, while the decondensed chromatin-containing nucleus of a vegetative cell controls processes connected to pollen tube germination and delivery of DNA to fertilize the egg and central cell [3,4]. Moreover, pollen is endowed by efficient mechanisms to eliminate “parasitic nucleic acids” like transposable elements [5] or viroid parasitic RNAs represented by pollen non-transmissible viroids, which are efficiently eradicated during pollen development and germination [6,7]. Viroids are plant pathogens that are highly stable due to the secondary structure of their single-stranded, circular RNA with lengths ranging within 239–401 nucleotides, which replicates autonomously in infected host plants. As a non-coding RNA [8,9], viroids possess thermodynamically stable or metastable structures important for their specific adaptation to the host metabolic machinery, suggesting intimate interaction of viroid parasitic RNAs with host generative tissues and pollen [10].

Citrus bark cracking viroid (CBCVd), apple fruit crinkle viroid (AFCVd) and potato spindle tuber viroid (PSTVd) are members of the family *Pospiviroidae*, where PSTVd is a type member of this family. CBCVd and AFCVd adapted recently to hop (*Humulus lupulus*) [11,12] and both are of high theoretical and practical interest. Viroids of the family *Pospiviroidae* are transcribed in an asymmetric rolling-circle mechanism by DNA-dependent RNA polymerase II (Pol II) of the host [13], possess a rod-like secondary structure and are localized primarily in the nucleolus. Viroid “parasitic” RNAs show fast evolutionary changes during the adaptation to new hosts [8,9]. The same is true for their spread and systemic propagation. For instance, some viroids are unable to reach floral organs [10]. Single RNA loops as parts of the viroid structure are important for their trafficking and propagation levels in different plant tissues including floral organs [14,15]. PSTVd adaptation to *N. tabacum* is mediated by a single mutation in loop E [16].

Vertical transmission via pollen and seeds determines the propagation of several viroids from parental plants to their progeny, while transmission of others is restricted [17]. In fact, horizontal viroid transmissibility shows wide quantitative variations depending on viroid-host combinations, viroid adaptation and environmental conditions, suggesting intimate interaction of viroid parasitic RNAs with host generative tissues [17,18,19,20,21,22,23], including the level of pathogenic changes caused by viroids in specialized pollen cells. It can be assumed in this respect that pollen- and seed-transmissible viroid species should be rather tolerant to pollen functions, while pollen non-transmissible variants could cause strong and specific pollen pathogenesis due to a lack of adaptation. However, despite the fact that most viroids induce pathogenic changes in plant somatic tissues, only a few data are available on possible distortions at the level of specialized male gametophyte [6,7,24].

In our previous work [7] we showed that AFCVd is pollen non-transmissible in the pollen-model plant *Nicotiana tabacum*, which is otherwise a rather tolerant and symptomless plant for this viroid. The elimination of AFCVd from pollen efficiently proceeds due to viroid degradation processes coupled with the low efficiency of native viroid replication in male gametophytes. In addition, this high elimination efficiency is not caused by a strong pathogenesis effect, because there were no significant changes in pollen vitality including in vitro germination ability [7]. Simultaneously, we showed by transcriptomics [25] that despite the low viroid pathogenicity, there are some complex changes in AFCVd-infected *N. tabacum* pollen. In our present work, we used the viroid-sensitive species *Nicotiana benthamiana* and showed that CBCVd especially caused significant changes in developing pollen collected from viroid transformants. Our results suggest that this pollen non-transmissible viroid, with elevated levels mediated by plant transformation, caused strong pathogenesis in pollen and significant developmental disorders in the plant reproduction process. Simultaneously, it shows that tolerance or metabolic adaptation is an important process for viroid transmissibility.

## 2. Results and Discussion

In our previous work, we selected *N. tabacum* as a model species to study the viroid elimination mechanism during pollen development [7]. While *N. tabacum* appeared to be an experimental host for AFCVd (Figure S1a), this species could not be experimentally infected with the second pollen non-transmissible hop viroid CBCVd (Figure S1b) [7]. Unlike *N. tabacum*, *N. benthamiana* is a host for both viroid isolates [26] and can be used to compare the elimination of both viroids during pollen development.

### 2.1. Flower and Pollen Developmental Stages in Nicotiana benthamiana

**Figure 1:**
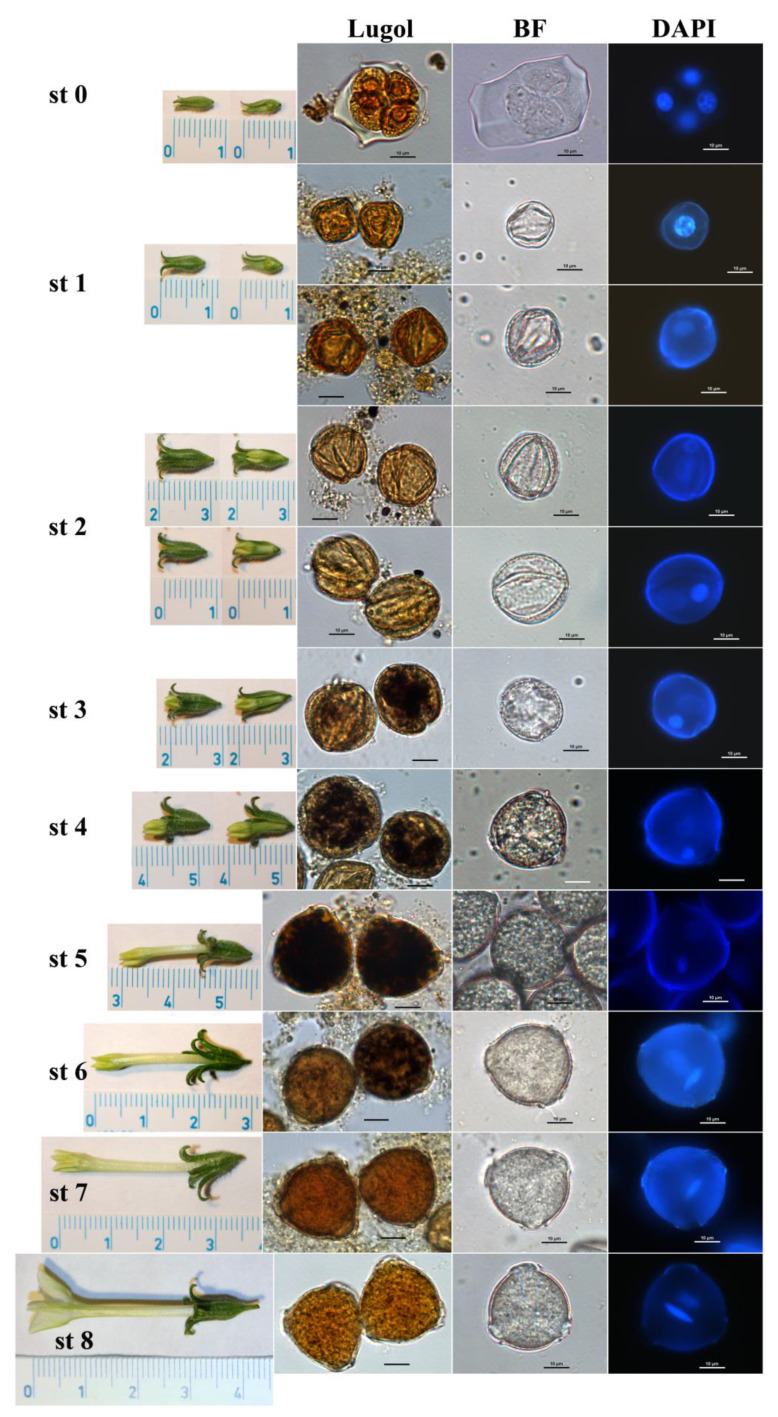
Images of *Nicotiana benthamiana* flower and pollen developmental stages (st 0–8), which were characterized according to length and morphology of flower buds, together with the cytological characteristics of pollen grains (number and shape of nuclei after DAPI staining, starch content after staining using Lugol solution). BF; pollen grains under bright field without any staining. Millimeter scale in flower bud images. Scale 10 µm in pollen microscopic images. A detailed word description of individual stages is given in Table S1.

In order to observe the elimination process during *N. benthamiana* pollen development, the individual pollen developmental stages were investigated using analysis similar to that developed for *N. tabacum* [27]. According to the length and morphology of flower buds (calix-corolla relationship), together with the cytological characteristics of pollen (number and shape of nuclei, starch content), several flower and pollen developmental stages were distinguished in *N. benthamiana* (Figure 1, Table S1).

The unicellular microspores (stage 1) can be found only in small flower buds with very small corolla (up to 5 mm in length) hidden inside the calix in *N. benthamiana* (Figure 1, Table S1). The first pollen mitosis, which microspores undergo and results in young immature bicellular pollen [28], occurs earlier in *N. benthamiana* compared to *N. tabacum*. While in *N. tabacum* the young immature bicellular pollen (stage 2) is present in the flower buds when corolla is appearing a few millimeters out of the calix [27], it occurs in flower buds with corolla still hidden inside the calix in *N. benthamiana*, but slightly longer flower buds compared to stage 1 (Figure 1, Table S1). Starch deposition is detectable histochemically inside the pollen when the corolla tip is clearly outside the calix (stage 3), reaches its maximum during stage 5, then decreases and it is not detectable histochemically in almost mature pollen in flowers shortly before anthesis (stage 7) in *N*. *benthamiana* (Figure 1, Table S1). *Nicotiana* genus belongs to bicellular pollen species [2] with a more diffused vegetative nucleus and compact generative nucleus when visualized under the microscope. The generative nucleus changes its shape during pollen maturation from round to spindle-shaped (Figure 1, Table S1) in both *Nicotiana* species.

### 2.2. Viroid Elimination during Pollen Development

**Figure 2:**
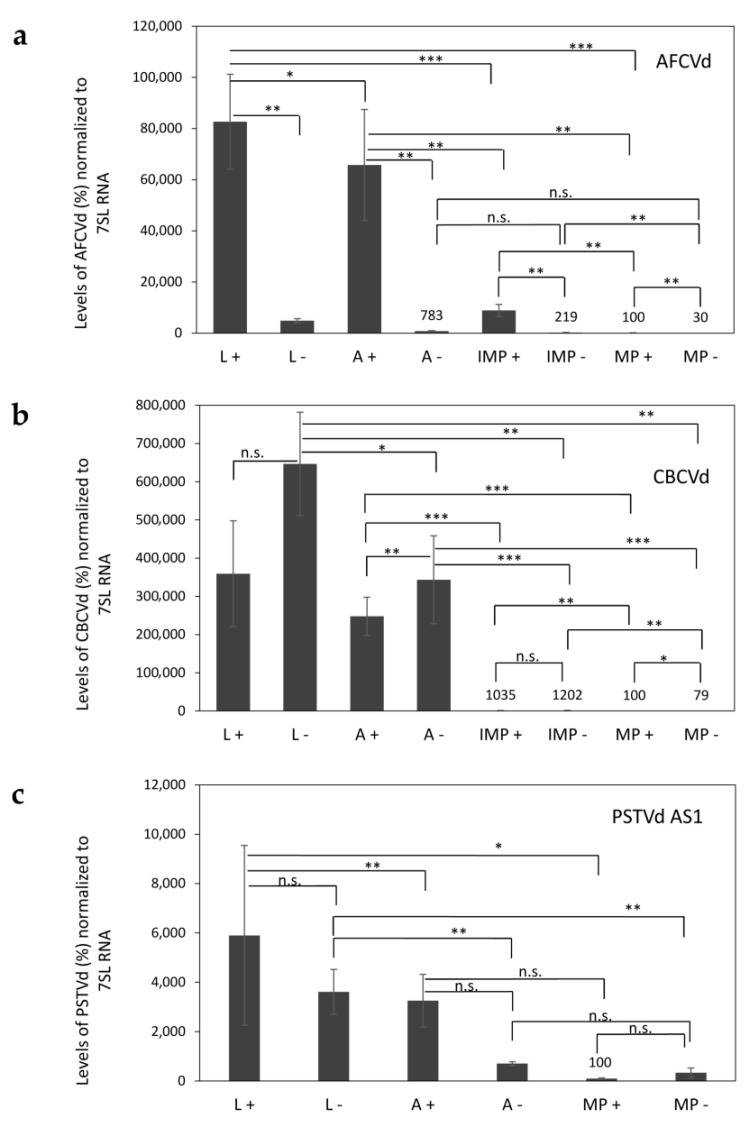
Relative levels of viroid (+) and (−) RNA chains in young leaves (L), immature anthers stages 3–4 (A), isolated immature pollen stages 3–4 (IMP) and mature pollen (MP) of *N*. *benthamiana* plants, which were inoculated by *A. tumefaciens* infiltration using infectious dimeric viroid cDNA-bearing plant vectors for (**a**) AFCVd, (**b**) CBCVd and (**c**) PSTVd AS1. Samples were collected 20 days p.i. and analyzed using ssRT-qPCR for relative levels of (+) and (−) chains. IMP was not collected from plants infected with PSTVd AS1. Healthy plants showed no qPCR signals of specific viroid. Each column represents the mean ± S.D. of two replicates of each PCR reaction. Lines with asterisks (* *p* < 0.1; ** *p* < 0.05; *** *p* < 0.01) indicate significant differences between connected values; (n.s.) statistically non-significant differences at *p* > 0.1. Remaining *p* values, which did not fit to the graph (**a**) AFCVd: L − against A − *p* = 0.0355; L − against IMP − *p* = 0.0092; L − against MP − *p* = 0.0083. (**b**) CBCVd: L + against A + *p* = 0.1005; L + against IMP + *p* = 0.041; L + against MP + *p* = 0.040.

During pollen development, both viroids, AFCVd and CBCVd, are strongly eliminated in infected *N*. *benthamiana* (Figure 2a,b). While in AFCVd-infected plants the (+) chains prevail significantly upon (−) chains in all studied tissues (Figure 2a), in CBCVd-infected plants more (−) chains were detectable in leaves and anthers (Figure 2b). The excess of (−) chains is an unusual feature for viroids but characteristic for CBCVd [7,26]. The relative levels and ratios of (+) and (−) chains in immature anthers (isolated from flower stages 3–4) are similar to leaves since intact anthers contain both sporophytic and gametophytic tissues. In contrast, a significant decrease of viroid levels was detected in immature pollen isolated from these anthers (approx. 7 and 200 times less for AFCVd and CBCVd, respectively) in comparison to whole anthers. Moreover, only traces of (+) viroid were detectable in mature pollen. The relative levels were approx. 800 and 3.6 × 10^3^ times lower for AFCVd and CBCVd, respectively, compared to leaves (Figure 2a,b). These results suggest a viroid elimination process similar to that detected in *N. tabacum* in our previous work [7]. The remaining traces of viroid in mature pollen were not detectable by molecular hybridization (data not shown).

**Figure 3:**
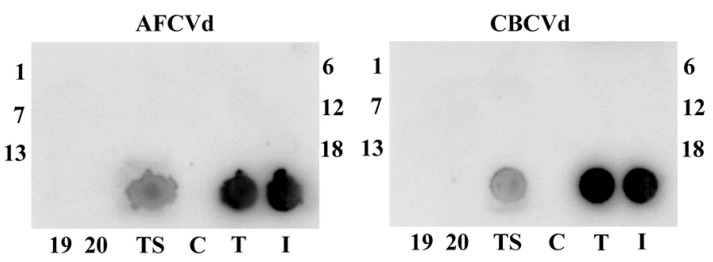
An example of dot-blot analysis of *N. benthamiana* offspring from AFCVd- or CBCVd-infected and transformed plants with infectious viroid cDNAs. Crude extracts from tissues of seedlings or upper leaves of flowering plants used as pollen sources were performed. Extract (25 μL) was dotted to nylon membrane and analyzed by hybridization to α^32^ P-labelled cDNA of either AFCVd or CBCVd probes. 1–20, extracts from individual three weeks old seedlings from seeds collected from *N. benthamiana* plants infected after *A. tumefaciens* infiltration (I). TS, mixed extracts from one-week-old seedlings from seeds collected from viroid cDNA-transformed *N. benthamiana* (T). Healthy control (C).

Neither AFCVd nor CBCVd were seed transmissible into *N. benthamiana* progeny after self-pollination, since no viroid infection developed in three-week-old *N. benthamiana* seedlings (Figure 3). This suggests no viroid transmission, neither through male nor female germlines. These results supplement and support our previous prediction about the non-transmissibility of CBCVd. Similarly to AFCVd, CBCVd is strongly eliminated from pollen in *Nicotiana* species due to specific degradation processes on the one hand and the reduction of viroid replication during pollen maturation on the other hand [7]. The processes of viroid elimination in pollen [7] could be similar to mechanisms induced by thermotherapy involving viroid degradation in somatic tissues, which is used for practical viroid eradication [29], however, further work is necessary to analyze such possibility.

We obtained similar results about AFCVd and CBCVd levels by the analysis of mature pollen from the infected hop. Furthermore, in these cases, only traces of viroid (+) and (−) chains were detectable in mature pollen in comparison to leaves (Figure S2). These results suggest that both viroids are practically pollen non-transmissible in hop similarly to another hop viroid, hop latent viroid (HLVd) [6,30]. Although, we did not check seed transmissibility of CBCVd and AFCVd in hop as native hosts in the present work, the pollen elimination of these two viroids could have positive practical consequences in hop selection and breeding programs, where viroid-free hybrid hop plants could be efficiently prepared by crossings like in the case of HLVd [30].

Unlike *N. tabacum*, *N. benthamiana* is also a native host for PSTVd. We have selected PSTVd for some comparative analyses because it is referred to as seed and pollen transmissible [20,31] in some species with wide variability. The rate of vertical transmission of PSTVd was found to be 0 to 90.2% in *Solanum lycopersicum* and 81% in *Petunia × hybrida* [32]. They used a PSTVd variant that was detected in cape gooseberry (*Physalis peruviana*) and that belongs to the ‘Oceanian’ variant [33]. We selected the highly pathogenic variant PSTVd AS1 that shows high RNA levels in *N. benthamiana* leaves [34]. We detected PSTVd AS1 by molecular hybridization in young leaves as well as in flower buds (stages 3–4) including corolla and stamens of *N. benthamiana* (Figure S3). This suggests that this viroid variant is transported to different tissues of *N. benthamiana* and that its trafficking is not blocked as in some other PSTVd variants [14].

PSTVd variant AS1 differs from the standard PSTVd variant Intermediate [35] by four mutations (G46C, C47U, U313A, U317C; see Figure S1d) at the border of terminal left and pathogenicity domain which fits to the severe symptoms induced by AS1 on *S. lycopersicum* cv Rutgers [34]. Loop 7 (U43·C318), which is required to be a cis Watson/Watson pair for systemic infection [36], is not mutated in AS1. This loop is usually closed by a G44·U317 pair but is a G44:C317 pair in AS1. This as a single mutation allows the mutant for local replication but not for systemic trafficking; however, additional mutations, as in the case of AS1, do compensate for this effect [37].

Our ssRT-qPCR analysis showed an excess of (+) chains over (−) chains in leaves and immature anthers (stages 3–4) of PSTVd AS1 infected plants. The levels of viroid RNA in immature anthers reached about 50% of that in leaves (Figure 2c). The average level of (+) chains was (59×) lower in mature pollen than in leaves. In addition, PSTVd AS1 clearly appeared to be seed non-transmissible after self-pollination (Figure S4). Our results suggest the involvement of a similar elimination process and/or depression of propagation in this species like in the case of AFCVd and CBCVd. However, we do not know, whether trafficking is also involved in this process, especially in blocking transmission via the female germline as described [15]. Our results are consistent with the findings that PSTVd exhibits very low seed transmissibility in some other plant species. For instance, 0.3% transmission was observed in *Capsicum annuum* var. grossum, 0.5% in *C. annuum* var. angulosum, 1.2% in *Glebionis coronaria* and no transmission in *Solanum melongena* or *Tagetes patula* [32]. In general, these findings suggest that seed transmissibility is rather a quantitative trait possibly depending on factors of viroid elimination, rate of propagation, protection [6,25] and trafficking in some species depending on structural elements as found for PSTVd-infected *N. benthamiana* [14,15].

### 2.3. Viroid Pathogenesis Levels in Naturally Infected and Viroid cDNA Transformed Pollen

In this study, we further concentrated on some aspects of viroid pathogenesis in pollen. On the one hand, it can be assumed that adaptation of pollen-transmissible viroid should not lead to significant disorders in pollen development and functions connected to pollination and fertilization. On the other hand, pollen non-transmissible viroid could show low or no adaptation to pollen metabolism to be transmitted and its increasing levels could induce some pathogenic changes or lethal developmental malfunctions.

A non-significant amount of pollen defects (aborted pollen up to 5%) were found in mature pollen of all naturally infected *N. benthamiana* (AFCVd, CBCVd and PSTVd AS1) in comparison to control plants using microscopy. However, a rare occurrence of mature pollen grains with three nuclei was noted in CBCVd-infected plants similarly to 35S:CBCVd transformed plants (see below in this section) despite the *Nicotiana* species naturally having bicellular mature pollen [2]. This suggests some specific metabolic regulatory changes caused by CBCVd in developing *N. benthamiana* pollen.

In our previous work [7] we also did not find any significant pathogenic changes in mature pollen phenotype or in pollen germination ability or any other obvious developmental distortions in AFCVd-infected *N. tabacum* plants using microscopy. So, we claimed that most probably the viroid elimination process is not connected to strong pollen pathogenesis. However, even the low level of AFCVd in pollen of infected *N. tabacum* plants, which was left upon the elimination process [7], caused some changes observed by transcriptome profiling and proteomic analyses [25]. These changes included genes involved in protein degradation, nuclear transport, phytohormone signaling, defense response and phosphorylation. Furthermore, several factors that might be involved in viroid elimination were identified to be differentially expressed; these included DNA-dependent RNA-polymerase, ribosomal proteins, Argonaute (AGO) proteins, nucleotide-binding proteins and RNA exonucleases [25].

**Figure 4:**
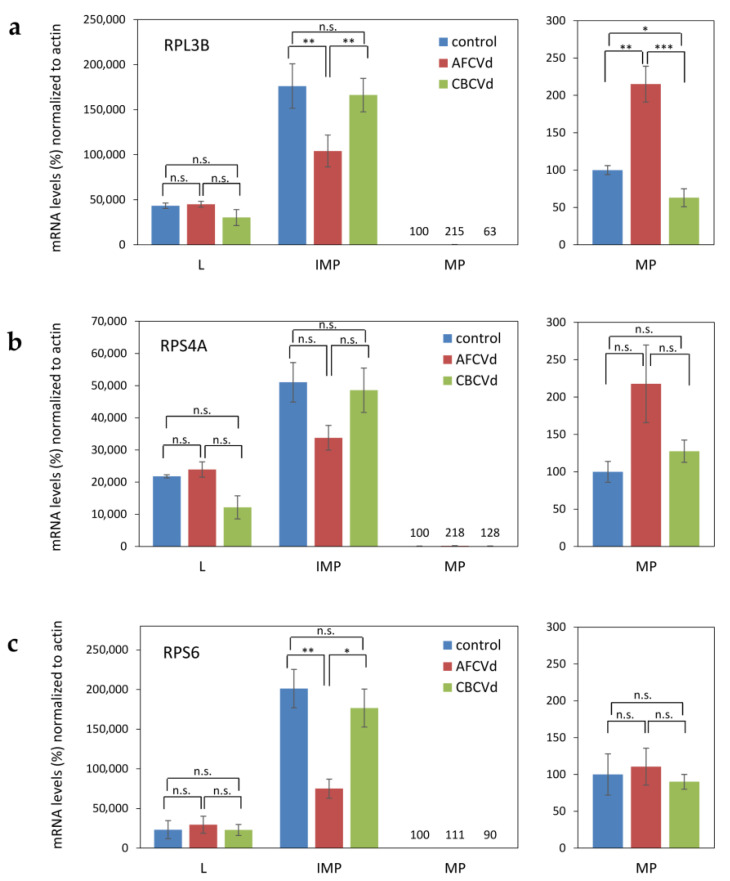
Relative levels of ribosomal protein mRNAs in healthy (control) and viroid infected (AFCVd and CBCVd) leaves (L), immature pollen (IMP) and mature pollen (MP) of *N. benthamiana*. In (**a**–**c**) individual ribosomal protein mRNAs are designated. To visualize the low levels of these mRNAs in MP, the respective parts are shown enlarged on the right side of each panel. Each column represents the mean ± S.D. of two replicates of each PCR reaction. Lines with asterisks (* *p* < 0.1; ** *p* < 0.05; *** *p* < 0.01) indicate statistically significant differences between connected values; (n.s.) statistically non-significant differences at *p* > 0.1.

Unlike *N. tabacum*, which is a rather tolerant species, *N. benthamiana* plants showed distinct morphological symptoms in response to both, CBCVd and AFCVd, infections (not shown). Especially stunting and reduction of the size of leaf blades depending on the condition of cultivation and time of inoculation as already presented [26]. In order to check for possible changes on the transcriptomic level in developing pollen of naturally infected AFCVd and CBCVd *N. benthamiana,* we selected a set of ribosomal protein genes homologs putatively important for sequestrome formation and mechanism of translational regulation [4,38] and used them as mRNA markers. Possible mRNA changes in immature pollen (stages 3–4) and mature pollen in comparison to leaves were analyzed by qPCR (Figure 4). There were differences especially in lower relative amounts of RPL3B, RPS4A and RPS6 mRNAs in AFCVd-infected immature pollen in comparison to healthy control, while the relative levels of these mRNAs were similar in leaves. In comparison higher levels of RPL3B and RPS4A were found in mature pollen of AFCVd-infected plants in comparison to control (Figure 4a,b). In the case of CBCVd, different results were obtained. CBCVd did not cause significant differences in the analyzed mRNAs in immature and mature pollen compared to control (Figure 4a–c). The different responses of these mRNA markers to AFCVd and CBCVd infection suggest that some of the changes caused by these viroids may be specific.

**Figure 5:**
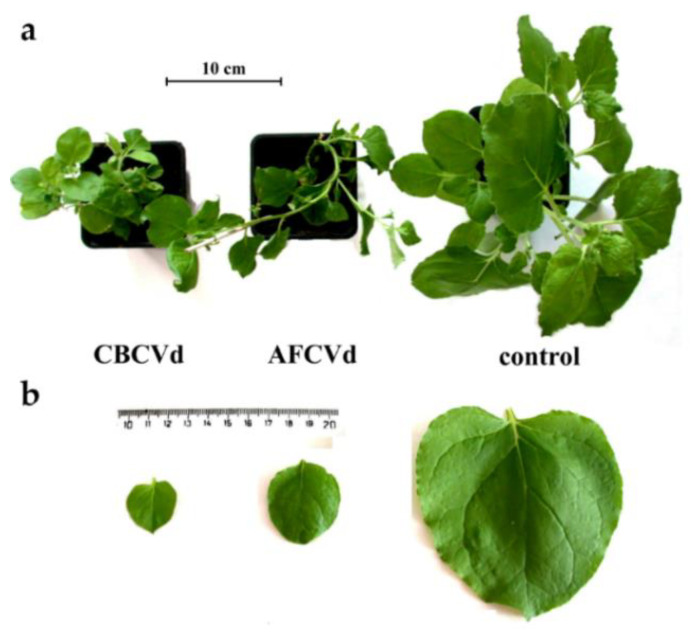
Growth characteristics of *N. benthamiana* transformed with CBCVd and AFCVd cDNAs under 35S promoter. (**a**) Overall plant habitus of CBCVd, AFCVd and control plants (view from above). (**b**) Size comparison of leaf blades of CBCVd, AFCVd and control plants. The fifth (expanded) leave counting from stem apex was collected from each variant.

**Figure 6:**
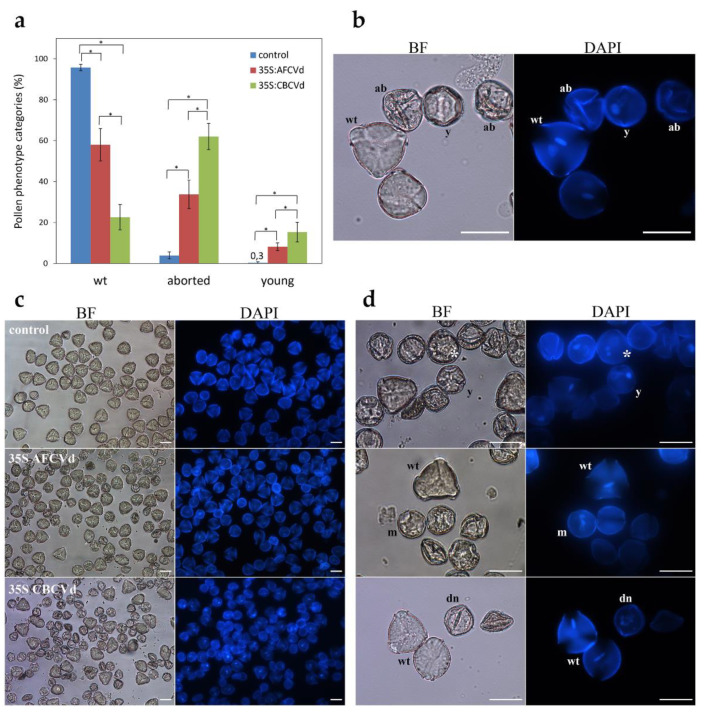
Microscopic analysis of mature pollen of control, 35S:AFCVd and 35S:CBCVd transformed *N. benthamiana*. (**a**) Quantification of the most common pollen phenotypes (in percent, mean ± S.D., *n* ≥ 9); healthy looking mature pollen grains (wt), aborted pollen (aborted) and young immature pollen grains (young). Lines with asterisks (* *p* < 0.05) indicate statistically significant differences between connected values. (**b**) Microscopic images of wt, aborted (ab) and young (y) pollen grains quantified in graph. (**c**) Overall microscopic views of mature pollen of the relevant variant. (**d**) Microscopic images of trinuclear pollen grain (asterisk), pollen with malformed nucleus (m) and pollen with partially degraded nucleus (dn) found in mature pollen of 35S:CBCVd transformed plants. (BF) bright field, (DAPI) fluorescence after DAPI staining and scale 30 µm in all microscopic images.

In our previous work with *N. tabacum,* we achieved CBCVd infection only by transformations with an infectious plant vector bearing dimeric viroid cDNA driven by 35S promoter, which led to approximately 10 times higher level of viroid RNA in mature pollen [7]. In the present work we used transformation of *N. benthamiana* with the same CBCVd-vector and an identically constructed AFCVd vector, named 35S:CBCVd and 35S:AFCVd, respectively, to study possibly enhanced viroid pathogenic effects. Ectopic expression of both viroids caused a dramatic reduction of overall plant habitus in comparison to control transformed with vector without viroid cDNAs (Figure 5a). The effect of CBCVd was more prominent than that of AFCVd, which can be visible e.g., on the sizes of leaf blades (Figure 5b). However, plants of both overexpression lines, 35S:CBCVd and 35S:AFCVd, were able to flower enabling detailed pollen analysis (Figure 6). A strong pathogenic effect of viroid overexpression in terms of an increase in pollen phenotype defects was found in these transformed plants. A high percentage of aborted pollen (34% and 62%) and a slight increase in young pollen grains (8% and 15%) were found in mature pollen of plants carrying 35S:AFCVd and 35S:CBCVd vector, respectively, in comparison to control plants with 3.9% of aborted pollen and 0.3% of young immature pollen (Figure 6a–c). Moreover, the rare occurrence of pollen with three nuclei (Figure 6d) like in naturally infected CBCVd *N. benthamiana*, but also pollen with malformed and/or degraded nuclei were monitored in 35S:CBCVd mature pollen (Figure 6d). A high percentage of pollen defects was connected with the reduced ability to self-pollinate because a minimum of ripe pods containing seeds was found in 35S:CBCVd maturing plants (data not shown). Further detailed studies (e.g., bright field microscopy, electron microscopy) of individual developmental anther and pollen stages are needed to give us a detailed view of which stages are influenced by viroid resulting in so many aborted pollen grains. In contrast to *N. benthamiana*, either 35S:AFCVd or 35S:CBCVd transformation did not cause a strong pathogenic effect in plant habitus and pollen of *N. tabacum* [7]. A much lower amount of aborted pollen and none of these pollen nuclei defects were found in *N. tabacum* [7]. It points to the different sensitivity of these two *Nicotiana* species to viroid in spite of both being hosts for AFCVd, but not for CBCVd in the case of *N. tabacum* [7,26]. In general, *N. tabacum* is considered to be a rather symptomless host plant to viroid, which is in accordance with our previous study [7].

Our results show that forcing expression of hop parasitic RNAs such as CBCVd and AFCVd, each of these seed non-transmissible viroids, can cause significant developmental distortions in the male gametophyte development of sensitive species, which can disturb pollination and plant fertilization. Our results suggest in general a process of evolutionary adaptation of pollen transmissible viroids to the metabolism of male gametophytes.

## 3. Materials and Methods

### 3.1. Plant Cultivation, Transformation and Viroid Inoculation

*Nicotiana benthamiana* plants were grown over the whole year in climate boxes at a temperature of 25 ± 3 °C with supplementary illumination [90 µmol m^−2^ s^−1^ PAR] to keep a 16 h day. For viroid inoculation and transformation of *N. benthamiana*, viroid cDNAs corresponding to AFCVd (GenBank AC AB104533/clone AK6-3), CBCVd (GenBank KM211547) [7,26] or PSTVd variant AS1 (GenBank AY518939) [34] were used (Figure S1). The plant vector with 35S promoter and particular dimeric viroid cDNA (Figure S5a–c) were infiltrated according to a protocol for transient expression [39]. *N. benthamiana* (designed as AFVCd, CBCVd and PSTVd infected plants) become infected as the expression of dimeric viroid cDNA promoted viroid replication pathway [7,39].

For plant transformation, the same plant vectors with dimeric AFCVd and CBCVd cDNAs under 35S promoter were used (Figure S5a,b [7]). *N. benthamiana* transformation was performed using the standard leaf disc method [40]. Regenerated transformed plants, designed as 35S:AFCVd and 35S:CBCVd, were maintained in a medium containing 100 mg/L kanamycin and 200 mg/L Timentin.

CBCVd infected hop pollen was collected in the middle of July 2017 from naturally infected male hop genotype 19058 grown in the experimental field of Slovenian Institute of Hop Research and Brewing. For AFCVd infection, male genotype from the crossing of female no. 211/96 x male no. 395/34 was obtained within the breeding and selection program in the Slovenian Institute of Hop Research and Brewing. Hop plants growing in the pots were AFCVd inoculated when having about 10 cm in height and forming up to three shoots using the biolistic method [26]. Each hop plant was inoculated five times in total with 250 ng DNA. Hop mature pollen was collected from AFCVd infected plants grown in the experimental field of the Institute of Plant Molecular Biology, Biology Centre of the Czech Academy of Sciences in the middle of July 2019.

### 3.2. Viroid Detection and Quantification

Viroids were detected from *N. benthamiana* plants three weeks post-infiltration. Dot-blot hybridization was performed as described [41] using crude RNA extracts from plant tissues in AMESS buffer (1 M sodium acetate pH 6.0 containing 1 M NaCl, 10 mM MgCl_2_, 20% ethanol and 3% SDS) prepared essentially as described [42]. Full-length CBCVd, AFCVd and PSTVd ^32^ P [dCTP]-labeled probes were used to check for CBCVd, AFCVd and PSTVd infections, respectively. Membranes from dot blots were scanned using the Typhoon PhosphoImager (Amersham Biosciences, Sunnyvale, CA, USA). The detection limit of this method is about 0.5 pg of viroid per dot [41].

For quantifications of parasitic RNAs, total RNA was isolated from 100 mg of leaves (a random mixture of cuttings from at least six young leaves), immature anthers, isolated immature pollen (both at stages 3–4, see Figure 1 and Table S1) and/or mature pollen (collected from open flowers after anther dehiscence) using CONCERT^TM^ reagent (Plant RNA Purification Reagent, Invitrogen) [7] and strand-specific viroid RT-qPCR (ssRT-qPCR) reactions were performed. This reaction enabled simultaneous RT-qPCR quantification of viroid (+) and (−) using Tth polymerase and primers CVdRTPL for reverse transcription of (+) and for CVdRTMI for reverse transcription of (−) chains following qPCR step, using PCR FOR and PCR REV primers [7,26] (Table S2). The quantification was performed on the CFX Connect^TM^ Real-Time PCR Detection System (Bio-Rad, Hercules, CA, USA) with Bio-Rad CFX Maestro qPCR software v. 1.1. Relative viroid levels were normalized with the “Delta-delta method” [43] to the level of 7SL RNA [26,34,44] as a reference marker. Because of the similar structure of 7SLRNA and viroid structures, the same RT-qPCR conditions with Tth polymerase were used for viroid RNA detection with the exception of using 7SL alpha and anti-beta primers (Table S2). To evaluate the efficiency of the qPCR reaction for each set of primers, five different concentrations of viroid DNA template were analyzed in duplicates to obtain a standard curve.

### 3.3. mRNA Quantification of Ribosomal Protein mRNAs as Markers

We used mRNA quantification of ribosomal proteins as markers to characterize viroid-initiated changes in *N. benthamiana* tissues. The quantification of mRNA levels was performed according to a published protocol [26] with the following modifications in annealing temperatures. The primer combinations listed in Table S2 for quantification of mRNA levels were derived based on *N. benthamiana* homologs of ribosomal proteins described in *N. tabacum* pollen (e.g., [38]). For analysis 1 μg of purified and DNase treated total RNA was reverse transcribed using oligo dT18 primer and Superscript III reverse transcriptase (Invitrogen, Carlsbad, CA, USA) at 50 °C for 60 min. RT-qPCR was then performed on the CFX Connect^TM^ Real-Time PCR Detection System (Bio-Rad, Hercules, CA, USA) using 20 μL of reaction mixture containing 5 μL of 20-fold diluted cDNA, 5 μL of 2 μM forward and reverse gene-specific primers (listed in Table S2) and 10 μL 2× SYBR green PCR master mix (Applied Biosystems, Waltham, MA, USA), under the following amplification condition: initial denaturation at 95 °C for 3 min, followed by 40 cycles of denaturation at 95 °C for 30 s, annealing temperature at 54 °C for 30 s, and extension at 72 °C for 35 s. At the end of the reaction, the specificity of each primer pair was assessed using a melting curve analysis. The products sizes were confirmed by melting analysis and 2% agarose gel electrophoresis. The abundance of a reference transcript of *Nt* Actin [7] was estimated in parallel in each sample. Ct values were measured using CFX Maestro qPCR software v. 1.1 (Bio-Rad). The relative values were standardized “Delta-delta method” and normalized to control mature pollen samples where the calibrator was set to 100%.

### 3.4. Pollen Developmental Stages Characterization

Nine flower and pollen developmental stages of wild type *N. benthamiana* plants were determined and characterized (see Figure 1 and Table S1) in relation to the length and morphology of flower buds (calix-corolla relationship), number and shape of nuclei and the presence of starch grains inspired by *N. tabacum* L. stages characterization [27]. Calix and corolla (after removing half of calix envelop) lengths of individual freshly collected flower buds were measured by a millimeter scale. Anthers were isolated manually from each flower bud. Pollen grains were released by gentle squeezing of anthers by tweezers to crack the anther walls directly into the buffer solution and immediately stained. The aliquot of pollen was stained with DAPI for cell nuclei visualization [7] and another aliquot of pollen was stained with Lugol’s staining solution (I_2_/KI stock solution: 2.5 g I_2_, 5 g KI, 200 mL water) for histochemical detection of starch grains. Inverted fluorescent microscope Nikon Eclipse TE2000-U was used for pollen grains observation in bright field and fluorescence (UV light) after DAPI staining. NIS-Elements^TM^ software (Nikon Imaging Software; v. 4.00) was used to capture images and include a scale.

### 3.5. Pollen Collection for Analysis

Mature pollen (MP) of *N. benthamiana* was collected daily from open flowers after anther dehiscence (flower stage 8, see Table S1) during at least one week when plants were intensively flowering. Pollen was weighed and stored at −20 °C until further analysis. Immature anthers (A) and/or immature pollen (IMP) were collected from fresh young flower buds of stages 3–4 together because of high tissue demand. IMP was isolated by gently cracking the anthers in prechilled mortar with 5% sucrose following filtration of released pollen suspensions and centrifugation [7]. Intact A and/or isolated IMP were weighted and stored at −80 °C until analysis.

### 3.6. Mature Pollen Grain Phenotypes

The aliquots of MP from healthy (control), viroid-infected and viroid-transformed *N. benthamiana* plants were resuspended in DAPI staining solution [7] to study the number and shape of nuclei (vegetative, generative) microscopically (see Section 3.4.). Three most common pollen phenotype categories were calculated: mature and late bicellular pollen with one diffuse vegetative nucleus and one spindle-shaped generative nucleus of more compacted structure (labeled as “wt”); aborted pollen of collapsed shape without any visible nuclei, only autofluorescence of exine (labeled as “ab”); young immature pollen with a diffuse vegetative nucleus and generative nucleus of spherical-round shape (labeled as “y”), which coincide visually with early bicellular pollen of wild type (control) plants. The percentage (mean ± S.D.) of individual pollen phenotype categories was calculated from at least 150 pollen grains per replicate and at least three replicates of infected and at least nine replicates of transformed variants. Other phenotypes (three nuclei pollen, pollen with malformed and/or degraded nuclei) were also monitored, but no percentage was calculated because of their rare occurrence.

### 3.7. Sequence Assembly and Structure Prediction of AFCVd, CBCVd and PSTVd

Viroid reference sequences of AFCVd (NC_003777), CBCVd (NC_003539) and PSTVd (NC_002030) from the Viral Genomes database [45,46] were used to search for further variants of each viroid via BLASTN [47]. A preliminary alignment of all sequence groups was produced via MAFFT L-INS-I [48]. Based on this alignment, incomplete sequences were removed; if necessary, the start and end of sequences were adjusted to standard positions, sequences were reverse complemented to (+)-strands, and redundant sequences were removed. Then a final alignment was produced via MAFFT X-INS-I [49] with options --maxiterate 1000 and --retree 100. For each of the final alignments, a consensus sequence and structure were calculated via CONSTRUCT [50] using RNAFOLD [51] with options -p -c (partition folding, circular RNA). Drawings of the consensus structures were done via R2R [52] (see Figure S1).

### 3.8. Statistical Evaluation

Statistical evaluation of pollen phenotypes was performed by Tukey-Kramer Multiple-Comparison Test using Number Cruncher Statistical System (NCSS 2004, Kaysville, UT, USA) after data transformation (square root) to meet the assumptions of normality and equal variance. Statistical evaluation of viroid RNAs and ribosomal protein mRNAs was performed by T-test using MS Excel (Microsoft Corp., Redmont, WA, USA).

## 4. Conclusions

Our results show that “forcing” the expression of hop parasitic RNAs such as CBCVd and AFCVd, each of these seed non-transmissible viroids, causes significant developmental distortions in male gametophyte development, which can disturb pollination and plant fertilization. Our results suggest in general a process of evolutionary adaptation of pollen-transmissible viroids to the metabolism of male gametophytes.

## Supplementary Materials

The following are available online at https://www.mdpi.com/article/10.3390/plants10112398/s1, Table S1: Detailed characterization of flower and pollen developmental stages according to length and morphology of flower buds (calix—corolla relationship) and the cytological characteristics of pollen grains (number and shape of nuclei, starch content) in *Nicotiana benthamiana*, Figure S1: Sequences and structures of AFCVd (GenBank LOCUS AB104533), CBCVd (GenBank LOCUS KM211547) and PSTVd variant AS1 (GenBank LOCUS AY518939) and PSTVd variant Int (Intermediate; GenBank LOCUS AY93719 or PTVA), Figure S2: Relative levels of viroid (+) and (−) RNA chains in leaves (L) and mature pollen (MP) of infected *H. lupulus*, Figure S3: Comparative detection of PSTVd AS1, Figure S4: Dot-blot hybridization analysis of viroid PSTVd AS1 seed transmissibility in *N. benthamiana*, Figure S5: Plant vector cassettes used for inoculation and transformation of *N. benthamiana*, Table S2: Primers used for strand-specific qRT-PCR and quantification of mRNA levels by qRT-PCR [53,54].

## References

[1] Hafidh S., Honys D. (2021). Reproduction Multitasking: The Male Gametophyte. Annu. Rev. Plant Biol..

[2] Brewbaker J.L. (1967). The distribution and phylogenetic significance of binucleate and trinucleate pollen grains in the Angiosperms. Amer. J. Bot..

[3] McCormick S. (1993). Male gametophyte development. Plant Cell.

[4] Hafidh S., Potešil D., Fíla J., Čapková V., Zdráhal Z., Honys D. (2016). Quantitative proteomics of the tobacco pollen tube secretome identifies novel pollen tube guidance proteins important for fertilization. Genome Biol..

[5] Slotkin R., Vaughn M., Borges F., Tanurdzi M., Becker J., Feijó J., Martienssen R. (2009). Epigenetic reprogramming and small RNA silencing of transposable elements in pollen. Cell.

[6] Matoušek J., Orctová L., Škopek J., Pešina K., Steger G. (2008). Elimination of hop latent viroid upon developmental activation of pollen nucleases. Biol. Chem..

[7] Matoušek J., Steinbachová L., Záveská Drábková L., Kocábek T., Potěšil D., Mishra A., Honys D., Steger G. (2020). Elimination of viroids from tobacco pollen involves a decrease in propagation rate and an increase of the degradation processes. Int. J. Mol. Sci..

[8] Diener T. (1995). Origin and evolution of viroids and viroid-like satellite RNAs. Virus Genes.

[9] Flores R., Hernández C., Martínez de Alba A.E., Daròs J.A., Di Serio F. (2005). Viroids and viroid-host interactions. Annu. Rev. Phytopathol..

[10] Venkataraman S., Badar U., Shoeb E., Hashim G., Abouhaidar M., Hefferon K. (2021). An inside look into biological miniatures: Molecular mechanisms of viroids. Int. J. Mol. Sci..

[11] Sano T., Mahaffee W., Pethybridge S., Gent D.H. (2009). Compendium of Hop Disease and Pests.

[12] Jakše J., Radišek S., Pokorn T., Matoušek J., Javornik B. (2015). Deep-sequencing revealed Citrus bark cracking viroid (CBCVd) as a highly aggressive pathogen on hop. Plant Pathol..

[13] Schindler I.M., Mühlbach H.P. (1992). Involvement of nuclear DNA-dependent RNA polymerases in potato spindle tuber viroid replication: A reevaluation. Plant Sci..

[14] Zhong X., Archual A.J., Amin A.A., Ding B. (2008). A genomic map of viroid RNA motifs critical for replication and systemic trafficking. Plant Cell.

[15] Jiang D., Wang M., Li S. (2017). Functional analysis of a viroid RNA motif mediating cell-to-cell movement in *Nicotiana benthamiana*. J. Gen. Virol..

[16] Wassenegger M., Spieker R.L., Thalmeir S., Riedel L., Sänger H.L. (1996). A single nucleotide substitution converts Potato spindle tuber viroid (PSTVd) from a noninfectious to an infectious RNA for *Nicotiana tabacum*. Virology.

[17] Matsushita Y., Yanagisawa H., Sano T. (2018). Vertical and horizontal transmission of Pospiviroids. Viruses.

[18] Desjardins P., Drake R., Atkins E., Bergh B. (1979). Pollen transmission of avocado sunblotch virus experimentally demonstrated. Calif. Agric..

[19] Kryczynski S., Paduch-Cichal E., Skrzeczkowski L. (1988). Transmission of three viroids through seed and pollen of tomato plants. J. Phytopathol..

[20] Singh R., Boucher A., Somerville T. (1992). Detection of potato spindle tuber viroid in the pollen and various parts of potato plant pollinated with viroid-infected pollen. Plant Dis..

[21] Mink G. (1993). Pollen and seed-transmitted viruses and viroids. Annu. Rev. Phytopathol..

[22] Pacumbaba E., Zelazny B., Orense J., Rillo E. (1994). Evidence for pollen and seed transmission of the coconut cadang-cadang viroid in *Cocos nucifera*. J. Phytopathol..

[23] Yanagisawa H., Sano T., Hase S., Matsushita Y. (2018). Influence of the terminal left domain on horizontal and vertical transmissions of tomato planta macho viroid and potato spindle tuber viroid through pollen. Virology.

[24] Castellano M., Martinez G., Marques M.C., Moreno-Romero J., Köhler C., Pallas V., Gomez G. (2016). Changes in the DNA methylation pattern of the host male gametophyte of viroid-infected cucumber plants. J. Exp. Bot..

[25] Shrestha A., Mishra A.K., Matoušek J., Steinbachová L., Potměšil D., Nath V.S., Awasthi P., Kocábek T., Jakše J., Záveská Drábková L. (2020). Integrated Proteo-Transcriptomic Analyses Reveal Insights into Regulation of Pollen Development Stages and Dynamics of Cellular Response to Apple Fruit Crinkle Viroid (AFCVd)-Infection in *Nicotiana tabacum*. Int. J. Mol. Sci..

[26] Matoušek J., Siglová K., Jakše J., Radišek S., Tsushima T., Brass J.R., Guček T., Duraisamy G., Sano T., Steger G. (2017). Propagation and some physiological effects of Citrus bark cracking viroid and Apple fruit crinkle viroid in multiple infected hop (*Humulus lupulus* L.). J. Plant Physiol..

[27] Tupý J., Süss J., Hrabětová E., Říhová L. (1983). Developmental changes in gene expression during pollen differentiation and maturation in *Nicotiana tabacum* L.. Biol. Plant..

[28] Honys D., Reňák D., Twell D., da Silva J.A.T. (2006). Male gametophyte development and function. Floriculture, Ornamental and Plant Biotechnology-Advances and Topical Issues.

[29] Matoušek J., Trněná L., Svoboda P., Oriniaková P., Lichtenstein C.P. (1995). The gradual reduction of viroid levels in hop mericlones following heat therapy: A possible role for a nuclease degrading dsRNA. Biol. Chem. Hoppe-Seyler.

[30] Matoušek J., Patzak J. (2000). A low transmissibility of hop latent viroid through a generative phase of *Humulus lupulus* L.. Biol. Plant..

[31] Fernow K.H., Peterson L.C., Plaisted R.L. (1970). Spindle tuber virus in seeds and pollen of infected potato plants. Am. Potato J..

[32] Matsushita Y., Tsuda S. (2016). Seed transmission of potato spindle tuber viroid, tomato chlorotic dwarf viroid, tomato apical stunt viroid, and Columnea latent viroid in horticultural plants. Eur. J. Plant Pathol..

[33] Verhoeven J.T.J., Botermans M., Roenhorst J.W., Westerhof J., Meekes E.T.M. (2009). First Report of Potato spindle tuber viroid in Cape Gooseberry (*Physalis peruviana*) from Turkey and Germany. Plant Dis..

[34] Matoušek J., Kozlová P., Orctová L., Schmitz A., Pešina K., Bannach O., Diermann D., Steger G., Riesner D. (2007). Accumulation of viroid-specific small RNAs and increase of nucleolytic activities linked to viroid-caused pathogenesis. Biol. Chem..

[35] Keese P., Symons R.H. (1985). Domains in viroids: Evidence of intermolecular RNA rearrangement and their contribution to viroid evolution. Proc. Natl. Acad. Sci. USA.

[36] Zhong X., Tao X., Stombaugh J., Leontis N., Ding B. (2007). Tertiary structure and function of an RNA motif required for plant vascular entry to initiate systemic trafficking. EMBO J..

[37] Wu J., Zhou C., Li J., Li C., Tao X., Leontis N.B., Zirbel C., Bisaro D.M., Ding B. (2020). Functional analysis reveals G/U pairs critical for replication and trafficking of an infectious non-coding viroid RNA. Nucl. Acids Res..

[38] Hafidh S., Potěšil D., Müller K., Fíla J., Michailidis C., Herrmannová A., Feciková J., Ischebeck T., Valášek L.S., Zdráhal Z. (2018). Dynamics of the Pollen Sequestrome Defined by Subcellular Coupled Omics. Plant Physiol..

[39] Matoušek J., Piernikarczyk R., Týcová A., Duraisamy G., Kocábek T., Steger G. (2015). Expression of SANT/HTH Myb mRNA, a plant morphogenesis-regulating transcription factor, changes due to viroid infection. J. Plant Physiol..

[40] Horsch R., Fry J., Hoffman N., Eichholtz D., Rogers S., Fraley R. (1985). A simple and general method for transferring genes into plants. Science.

[41] Matoušek J., Schröder A.R.W., Trněná L., Reimers M., Baumstark T., Dědič P., Vlasák J., Becker I., Kreuzaler F., Fladung M. (1994). Inhibition of viroid infection by antisense RNA expression in transgenic plants. Biol. Chem. Hoppe-Seyler.

[42] Palukaitis P., Cotts S., Zaitlin M. (1985). Detection and identification of viroids and viral nucleic acids by “dot-blot” hybridization. Acta Hortic..

[43] Pfaffl M. (2001). A new mathematical model for relative quantification in real-time RT-PCR. Nucleic Acids Res..

[44] Matoušek J., Junker V., Vrba L., Schubert J., Patzak J., Steger G. (1999). Molecular characterization and genome organization of 7 SL RNA genes from hop (*Humulus lupulus* L.). Gene.

[45] Viral Genome Browser (Pospiviroidae). https://www.ncbi.nlm.nih.gov/genomes/GenomesGroup.cgi?taxid=185751.

[46] Brister J.R., Ako-Adjei D., Bao Y., Blinkova O. (2015). NCBI viral genomes resource. Nucleic Acids Res..

[47] Morgulis A., Coulouris G., Raytselis Y., Madden T., Agarwala R., Schäffer A. (2008). Database indexing for production MegaBLAST searches. Bioinformatics.

[48] Katoh K., Rozewicki J., Yamada K. (2017). MAFFT online service: Multiple sequence alignment, interactive sequence choice and visualization. Brief. Bioinform..

[49] Katoh K., Toh H. (2008). Improved accuracy of multiple ncRNA alignment by incorporating structural information into a MAFFT-based framework. BMC Bioinform..

[50] Wilm A., Linnenbrink K., Steger G. (2008). ConStruct: Improved construction of RNA consensus structures. BMC Bioinform..

[51] Gruber A., Lorenz R., Bernhart S., Neuböck R., Hofacker I. (2008). The Vienna RNA websuite. Nucleic Acids Res..

[52] Weinberg Z., Breaker R. (2011). R2R–software to speed the depiction of aesthetic consensus RNA secondary structures. BMC Bioinform..

[53] Matoušek J., Kocábek T., Patzak J., Bříza J., Siglová K., Mishra A., Duraisamy G., Týcová A., Ono E., Krofta K. (2016). The “putative” role of transcription factors from HlWRKY family in the regulation of the final steps of prenylflavonid and bitter acids biosynthesis in hop (*Humulus lupulus* L.). Plant Mol. Biol..

[54] Matoušek J., Stehlík J., Procházková J., Orctová L., Wullenweber J., Füssy Z., Kováčik J., Duraisamy G.S., Ziegler A., Schubert J. (2012). Biological and molecular analysis of the pathogenic variant C3 of potato spindle tuber viroid (PSTVd) evolved during adaptation to chamomile (*Matricaria chamomilla*). Biol. Chem..

