# Supplementary material for: Transformation of Seed Non-Transmissible Hop Viroids in Nicotiana benthamiana Causes Distortions in Male Gametophyte Development
Source: Plants (Basel). 2021 Nov 6;10(11):2398. doi: 10.3390/plants10112398 (PMC8624972; doi:10.3390/plants10112398)
Supplement: Supplementary file 1 [file plants-10-02398-s001.zip › plants-1410911-supplementary.pdf]

plants

MDPI

Supplementary Materials: Transformation of Seed Non-transmissible Hop Viroids in *Nicotiana benthamiana* Causes Distortions in Male Gametophyte Development

Lenka Steinbachová [1], Jaroslav Matoušek [2], Gerhard Steger [3], Helena Matoušková [2], Sebastjan Radišek [4] and David Honys [1,*]

Table S1. Detailed characterization of flower and pollen developmental stages according to length and morphology of flower buds (calix - corolla relationship) and the cytological characteristics of pollen grains (number and shape of nuclei, starch content) in *Nicotiana benthamiana*.

Stage	Length of flower buds (mm)	Detail description of flower buds	Cytological characteristics of pollen developmental stage
0	6–7	Corolla (2–2.5 mm) hidden inside the calix	Tetrads of four haploid uninuclear microspores enclosed within callose wall. Starch grains are not detectable histochemically inside the tetrads.
1	7.5–9	Corolla (2.5–3 mm) hidden inside the calix	Microspores with central position of nucleus. Starch grains are not detectable histochemically.
		Corolla (3–5 mm) hidden inside the calix	Polarized microspores before first pollen mitosis. Starch grains are not detectable histochemically inside the microspores, but were released from disrupted anther tissues.
2	8.5–11	Corolla (5–9 mm) still hidden inside the calix	Immature bicellular pollen grains with diffused vegetative nucleus and compact rounded generative nucleus. Starch grains are not detectable histochemically.
		Corolla tip simultaneously with calix	
3	9–11.5	Corrolla tip out of the calix	Immature bicellular pollen grains with vegetative and generative nuclei of round shape. Starch is clearly detectable histochemically.
4	11.5–14	Corolla head out of calix, tubular part of corolla is still hidden inside the calix	Immature bicellular pollen grains with vegetative and generative nuclei of round shape. Accumulation of starch further increases.
5	20–25	Tubular part of corolla visible out of calix	Immature bicellular pollen grains are full of starch, generative nucleus starts to elongate.
6	29–31	Tubular part of corolla growing in length	Immature bicellular pollen grains with generative nucleus of elongated shape, decreasing content of starch.
7	ca. 35–38	Flower shortly before opening with intact anthers	Almost mature pollen grains, generative nucleus is spindle-shaped, starch is not detectable histochemically.
8	ca. 38–41	Partially or fully open flower with cracked anthers	Mature pollen grains, generative nucleus is spindle-shaped, starch is not detectable histochemically.

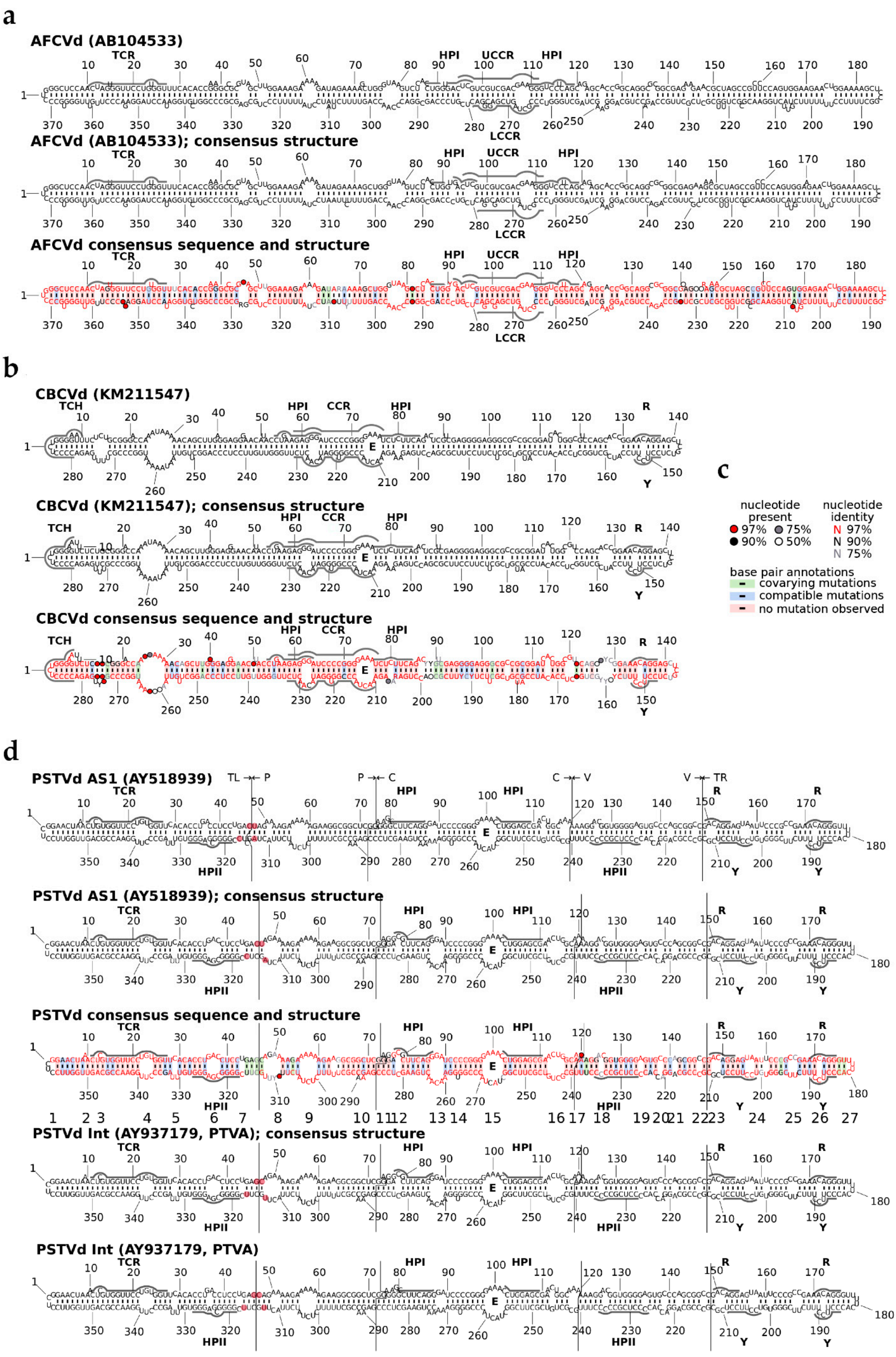
a
AFCVd (AB104533)
AFCVd (AB104533); consensus structure
AFCVd consensus sequence and structure
TCR
HPI
UCCR
LCCR
b
CBCVd (KM211547)
CBCVd (KM211547); consensus structure
CBCVd consensus sequence and structure
TCH
CCR
R
Y
c
nucleotide present
97%
75%
90%
50%
nucleotide identity
N 97%
N 90%
N 75%
base pair annotations
covarying mutations
compatible mutations
no mutation observed
d
PSTVd AS1 (AY518939)
PSTVd AS1 (AY518939); consensus structure
PSTVd consensus sequence and structure
PSTVd Int (AY937179, PTVA); consensus structure
PSTVd Int (AY937179, PTVA)
TL
P
C
V
TR
HPII
E
1 2 3 4 5 6 7 8 9 10 11 12 13 14 15 16 17 18 19 20 21 22 23 24 25 26 27

Figure S1. Sequences and structures of (a) AFCVd (GenBank LOCUS AB104533), (b) CBCVd (GenBank LOCUS KM211547) and (d) (PSTVd variant AS1 (GenBank LOCUS AY518939) and PSTVd variant Int (Intermediate; GenBank LOCUS AY93719 or PTVA). (a), (b) Top: Sequence with thermodynamically optimal structure. Middle: Sequence with consensus structure. Bottom: Consensus sequence and consensus structure in all viroids and variants. (c) Color code used for the annotation of nucleotides and base pairs in the consensus sequences and structures. (d) From top to bottom: Sequence of PSTVd variant AS1 with individual structure; sequence of PSTVd AS1 with consensus structure; PSTVd consensus sequence and structure (loop numbering is given below the structure according to [14]); sequence of PSTVd variant Intermediate (Int) with consensus structure; sequence of PSTVd Int with individual structure. Terminal Conserved Region (TCR); Upper and Lower Central Conserved Region (UCCR and LCCR, respectively); Hairpin I, II (HPI, HPII); Terminal Conserved Helix (TCH); RY motif (a recognition site of viroid-binding protein Virp1 [9]); Terminal Left domain (TL); Pathogenicity domain (P); Variable domain (V); Conserved region (C).

a

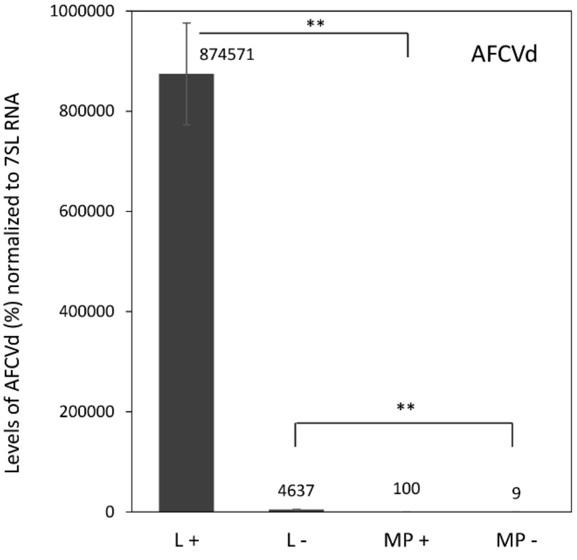

b

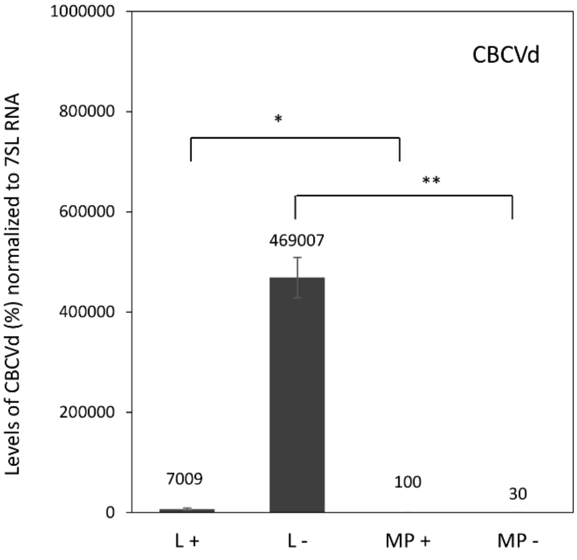

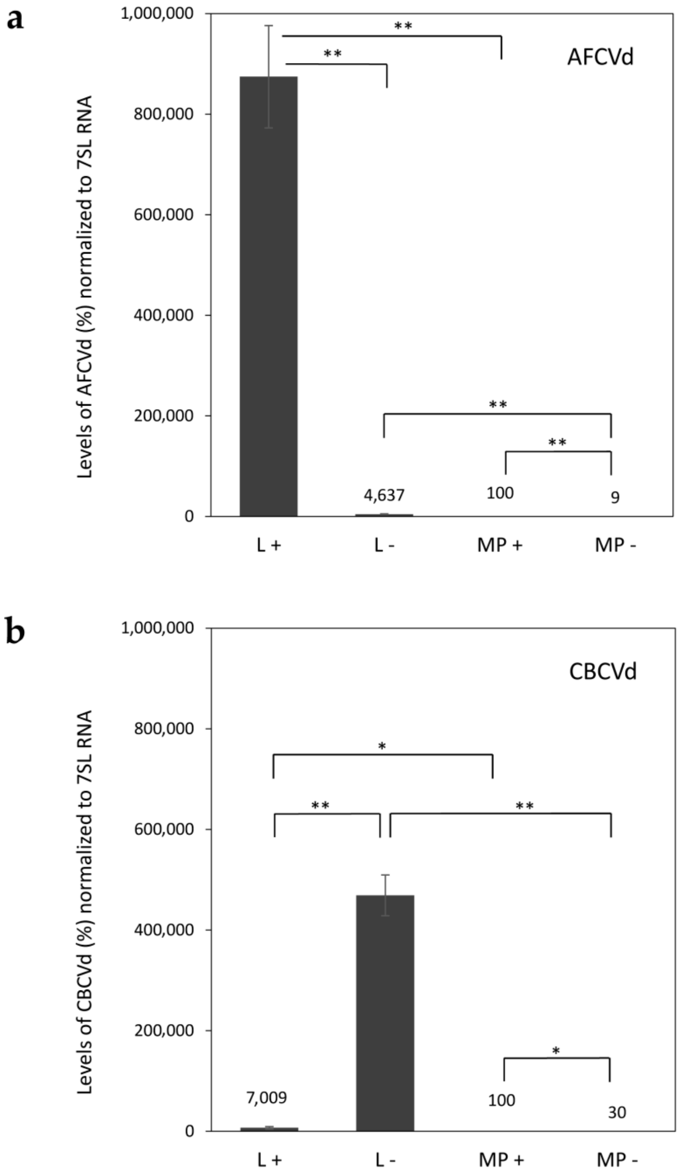

Figure S2. Relative levels of viroid (+) and (–) RNA chains in leaves (L) and mature pollen (MP) of infected *H. lupulus*. Samples were collected upon flowering of infected hop male plants after either biolistically inoculated AFCVd (**a**) or natively infected CBCVd (**b**) in the middle of July of the seasons 2017 and 2019, respectively. RNA was isolated and ssRT-qPCR was performed as described in Material and Methods. The levels of (+) chains in MP were set to 100%. No signal was observed from pollen of AFCVd and CBCVd negative control hop plants. Each column represents the mean ± S.D. of two replicates of each PCR reaction. Lines with asterisks (*, p < 0.1; **, p < 0.05) indicate significant differences between connected values.

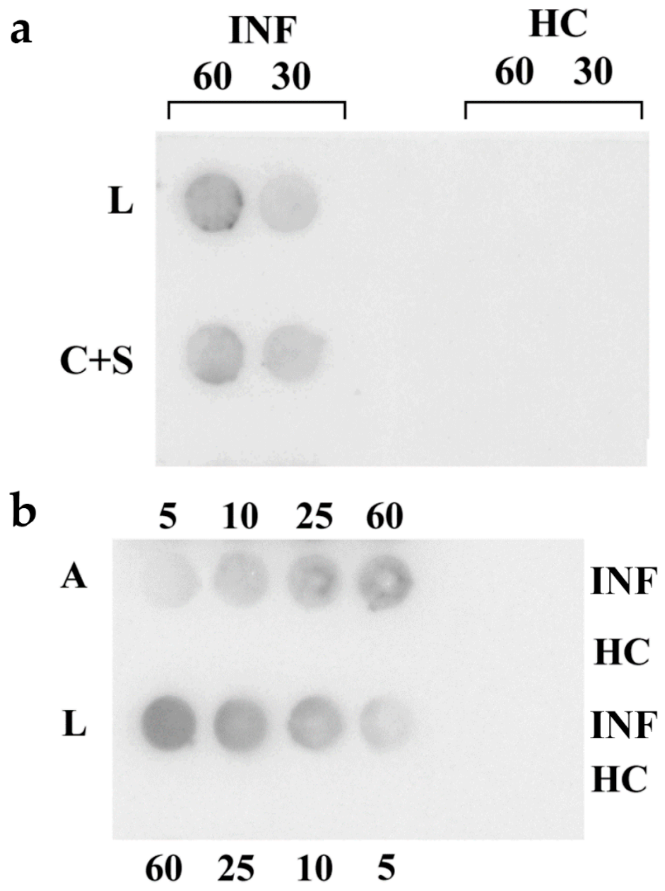

Figure S3. Comparative detection of PSTVd AS1. (**a**) Comparative detection of PSTVd AS1 in young upper leaves (L) and in young flowers (stage 3-4) including corolla and stamens (C+S) of infected (INF) and healthy control (HC) *N. benthamiana* plants. Tissues were prepared and extracted with AMESS buffer using standard protocol (Material and Methods). 60 and 30 µl of extracts were dotted to nylon membrane and hybridized against AS1 PSTVd α^{32}P dCTP-labelled cDNA probe. (**b**) Comparative analysis of PSTVd levels using isolated RNA from anthers (A) and leaves (L) of infected (INF) and healthy control (HC) plants of *N. benthamiana*. RNA was isolated using plant reagent, 20 µg were dissolved in 100 µl of denaturation solution (see Material and Methods), denatured and spotted to nylon membrane in indicated amounts (5, 10, 25, 60 µl) and hybridized to PSTVd AS1 cDNA probe.

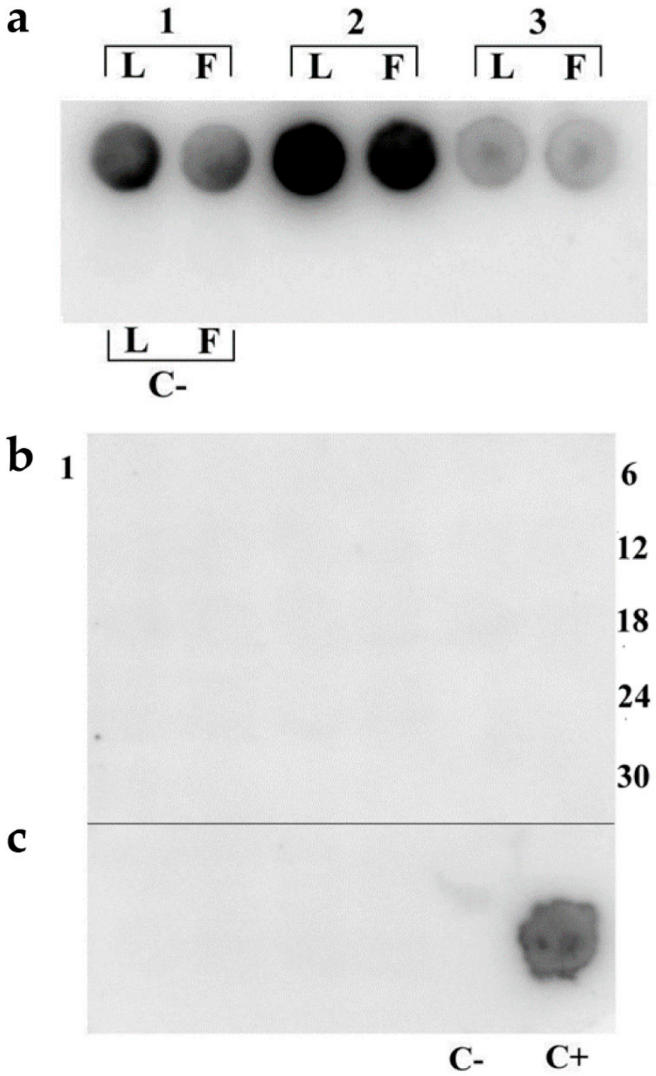

Figure S4. Dot-blot hybridization analysis of viroid PSTVd AS1 seed transmissibility in *N. benthamiana*. (**a**) radioactive signals from upper young leaves (L) or immature flowers (F, stages 3-4) from viroid-infected plants No 1-3. (**b**) 1-30, extracts from thirty-four weeks old individual seedlings - progeny of *N. benthamiana* plant No 2 from panel (**a**) after self-pollination. (**a**), (**c**) negative (C-) and positive (C+) controls. Plant tissues were extracted with AMESS extraction [7]. Extracts (25 µl) were dotted to nylon membrane and analyzed by hybridization to α^{32}P-labelled cDNA of PSTVd AS1 used as a probe.

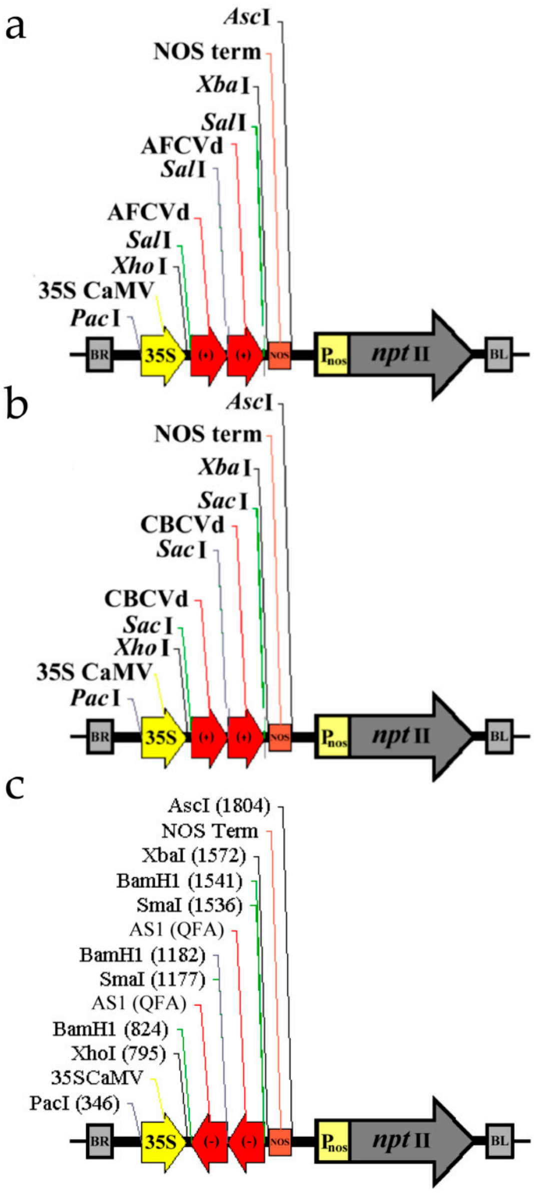

Figure S5. Plant vector cassettes used for inoculation and transformation of *N. benthamiana*. AFCVd (a), CBCVd (b) [26] and PSTVd (c) [39] infectious dimeric cDNAs integrated in modified expression cassettes containing CaMV 35S promoter and NOS terminator that were constructed and cloned via PacI and AscI to plant vector pLV07 previously [7, 53].

Table S2. Primers used for strand-specific qRT-PCR and quantification of mRNA levels by qRT-PCR.

Designation	Sequence 5′→ 3′	Purpose	Reference
	**PSTVd AS1**		
RT(+)	GGATCCCTGAAGCGCTCCTCCG	cDNA synthesis – reverse transcription	[54]
RT(-)	GGATCCCCGGGGAAACCTGGAG		
PCRI	CTCGGGAGCTTCAGTTGTTTC	qRT-PCR	[54]
PCRI	CTTTCTTCGGGTGTCCTTCC		
	**AFCVd**		
AFCVdRTPL	TCGTCGACGACGAGTCACCAGGTG	cDNA synthesis – reverse transcription	[7,26]
AFCVdRTMI	GTGACTCGTCGTCGACGAAGGGTC		
AFCVd PCR FOR	CCGGTCGTGGATACCTAGGA	qRT-PCR	[7,26]
AFCVd PCR REV	ACGCGGCCTTCGGTGTG		
	**CBCVd**		
CVdRTPL	AAGCCTGGGAGGAACAACCCAAGAG	cDNA synthesis – reverse transcription	[7,26]
CVdRTMI	GGATCCCCGGGGAAATCTCTTCAG		
CVd PCR FOR	TCACTGGCGTCCAGCACC	qRT-PCR	[7,26]
CVd PCR REV	AGGAAGAAGCGACGATCGG		
	**7SL RNA**		
primer α	TGTAACCCAAGTGGGGG	normalization	[44]
anti-β	GCACCGGCCCGTTATCC		
	**Ribosomal proteins RPS4A (Niben101Scf14717g00010.1)**		
S4AF	CATTATCTTGCGGAACAGGTTG	qRT-PCR	-
S4AR	CGGACCTTGCAAAGCTTAAAC		
	**RPS6 (Niben101Scf03584g01006.1)**		
S6F	CGAGTTCGTCTTCTGCTGC	qRT-PCR	-
S6R	TTCCTTGGAAAGGTTGAAGAG		
	**RPL3B (Niben101Scf02802g00009.1)**		
L3BF	GCCCATTTGATGGAGATCC	qRT-PCR	-
L3BR	TGCCAGGCACCAATACAAG		
	**Nt Actin**		
ACT-F1	TTCTGTTCCAACCATCAATGA	qRT-PCR	[7]
ACT-R1	GTACCACCACTGAGGACAATGT		
